# Nondominant hand computer mouse training and the bilateral transfer effect to the dominant hand

**DOI:** 10.1038/s41598-021-83770-4

**Published:** 2021-02-18

**Authors:** Drew Schweiger, Richard Stone, Ulrike Genschel

**Affiliations:** 1 Department of Industrial and Manufacturing Systems Engineering, Iowa State University, Ames, IA 50011 USA; 2 Department of Statistics, Iowa State University, Ames, IA 50011 USA

**Keywords:** Risk factors, Engineering

## Abstract

This study explored the effects of training computer mouse use in the nondominant hand on clicking performance of the dominant and nondominant hands. Computer mouse use is a daily operation in the workplace and requires minute hand and wrist movements developed and refined through practice and training for many years. Our study had eleven right-handed computer mouse users train their nondominant hand for 15 min a day, five days per week, for six weeks. This study found improved performance with the computer mouse in the dominant hand following nondominant hand training because of the bilateral transfer effect of training. Additionally, our study showed that the nondominant hand is capable of learning the complex movements that our dominant hand has trained for many years. Last, our research showed that nondominant hand performance decreases when the skill is not trained for over a year, but the performance is significantly higher than that prior to the original training and can be rapidly relearned. Overall, training the nondominant hand on the computer mouse will allow for improved performance in industry while allowing safer, sustainable, and more achievable work in a multitude of economies.

## Introduction

Dominant hand preference is formed in early childhood and continually trained throughout one’s lifetime. People reinforce dominant hand preference when performing tasks with the same hand by building muscle memory and becoming more accustomed and comfortable having always done tasks a certain way. Many tools and human interfaces are designed for people who are predominantly right-handed, which further promotes the usage of a single hand for the majority of work functions. The formation of hand dominance, known as handedness, becomes apparent between the ages of seven and nine months when children begin repeatedly reaching and grabbing objects with a single hand^1^. All tasks, such as using a computer mouse, eating, kicking, leaning, throwing, and writing, have a preferred dominant side that is not always the same. A person may complete many tasks with their left hand while using a computer mouse in their right hand. Thus, handedness is often task specific, and tasks performed daily form a much stronger handedness than those performed only a few times a year^2^. The dominant hand continues to be used throughout adulthood as tasks become instinctual, thereby optimizing task processing time and performance. If a dominant hand is not formed, the performance of otherwise simple work tasks can become cumbersome, and time is lost due to the amount of time spent thinking over which hand should be used^2^. As humans age into their 50 s, there remains a large prevalence of hand dominance in regard to finger and wrist speed and agility^3^. However, as people age into their 60 s and 70 s, there begins to be a reduction in hand superiority as the body begins to rebalance performance with each hand^3^.

Further research on hand usage has affirmed an association between hand dominance and injury. A right-hand dominant person is estimated to be five times more likely to develop carpal tunnel syndrome in their right hand than in their left^4^, and people who use the computer mouse with the same hand throughout the day have significantly more symptoms of injury in the mouse-side hand than in the hand on the non-mouse side^5^. Another study found that in 83% of people who suffered a hand injury, the dominant hand was affected, and an upper extremity disorder of the hand was more likely to occur in those who injured their dominant hand than in those who injured their nondominant hand^6^. Once the hand is injured, the dominant hand was shown to have significantly higher erosion, joint space narrowing, and damage progression than an injured nondominant hand^7^. After surgery, both hands have significantly less pain, but the prevalence of pain reported after surgery is higher for those with surgery on the dominant hand versus the nondominant hand^8,9^.

In today’s society, computers are used daily by the majority of the population, and most people have a lifetime of experience performing mouse and keyboard tasks. Workstations are set up with the mouse on the right, leading to consistent use of the mouse in people’s right hand and the formation of a hand dominance. When employees consistently operate the mouse with the same hand over many years, they can develop repetitive use injuries, such as carpal tunnel syndrome, tendonitis, arthritis, or other musculoskeletal injuries to the hand, which can hinder their ability to work^10^. Carpal tunnel syndrome occurs from compression of the median nerve caused by repetitive wrist movements in extension or flexion^11^. The prevalence of carpal tunnel injuries has been widely researched, with a prevalence between 3.8 and 7.8% of the workforce, and these injuries occur more frequently in the dominant hand^12–14^. Repetitive blue-collar work and white-collar work, such as continually driving screws or performing data entry tasks, have a higher prevalence of carpal tunnel syndrome than any other occupation^15^. While workplace-related injuries can occur in either hand, they are more prevalent in the dominant hand and often lead to companies paying workers’ compensation claims, along with the additional recruiting, onboarding, and training costs incurred by companies to fill temporary positions. These measures can be costly to companies as there are “500,000 carpal tunnel releases costing over $2 billion performed each year in the United States”^16^.

To reduce dominant hand injuries, this study seeks to examine performance related to hand dominance for computer use and assess whether the nondominant hand can be trained to perform at a level similar to the dominant hand. If the nondominant hand can attain the same level of efficiency as the dominant hand, this will allow for a more balanced use of both hands, thus decreasing the risk of injury in the dominant hand due to overuse. In the context of an office environment, the existing literature has not addressed whether the level of proficiency of the dominant hand is achievable by the nondominant hand through adequate training of the nondominant hand. Additionally, the literature has not addressed the learning curve related to the nondominant hand use of a computer mouse and the time it takes to reach the same level of proficiency as observed with the dominant hand. Learning curves are important tools for analyzing the relationship between performance and a period of training or learning a task^17^. Visually, learning curves traditionally show an immediate improvement as the learner becomes more familiar with the task^17^. However, there are natural limits that people reach in their performance, which is exhibited by a leveling off in the curve^17^. Learning curves are affected by many factors, making them very task specific^18^, and this study assesses the learning curve associated with the nondominant hand use of the computer mouse. Research has shown that there is an ability to improve performance of the nondominant hand for a variety of tasks. Training with chopsticks for 30 days in experienced users’ nondominant hand for 30 min a day resulted in significant improvement in the smoothness and speed of the nondominant hand when utilizing chopsticks^19^. Additionally, ten days of training the nondominant hand in precision drawing tasks showed significant improvements in smoothness and speed of the nondominant hand in carrying out the task^20^. After fifteen days of training, the nondominant writing hand demonstrated an improvement that more closely represented performance with the dominant hand^21^. Basic everyday tasks such as using chopsticks (in countries for which this is the primary utensil), drawing, and writing are hand movement skills that have been trained over a lifetime. Importantly, each of these skills demonstrated performance improvements after training the nondominant hand. Similar to previous research, a component of this study was to determine if nondominant hand improvements can occur with the use of a computer mouse for its workplace applications and to identify the time it takes for the nondominant hand to approach the capability of the dominant hand. We hypothesized that the nondominant hand will perform as well as the dominant hand originally performed after training for six weeks.

An additional objective for this study was to examine whether training the nondominant hand on a computer mouse can improve the performance of the dominant hand on the same task. Many companies are consistently looking at performance metrics to increase production by improving the efficiency of their employee’s workflow. Regarding tasks that are completed on a computer, this may entail reducing the number of entries needed or rearranging the position of icons to help reduce time spent on the computer. Eventually, the changes to the user interface and number of entries can no longer be reduced. However, the current literature does not address whether industry performance is limited due to failure to train our nondominant hand or whether industry performance of the dominant hand can increase by training the nondominant hand. Bilateral transfer is the phenomenon where training one side of the body can lead to improvements with the other side of the body^22^. Bilateral transfer of learning has been observed in a variety of applications with upper and lower extremities from training the nondominant limbs. Soccer players who trained only their nondominant leg for eight weeks performed significantly better in a variety of soccer tasks with both their nondominant and dominant legs than those who continued to train normally^23^. Long jump athletes who trained their nondominant leg for twelve weeks, two days a week, for 1.5 h a day saw a significant increase in jumping performance compared with those who trained only their dominant leg^24^. Additionally, fencers who trained the nondominant hand for six weeks, five days a week, for 30 min a day, showed improved performance with the dominant hand beyond that of participants who trained only their dominant hand^25^. Studies outside of sports applications have shown bilateral transfer to occur in the hand when training different aiming, steadiness, and finger tapping exercises with the nondominant hand, as there were significant decreases in time for both the trained and untrained hands^26^. We hypothesized that people can train with a computer mouse in their nondominant hand and see improvements in performance with their dominant hand because of the bilateral transfer of learning. Training the nondominant hand could help to remove the natural stagnation that people develop in their computer performance.

To further support our hypotheses, we also examined the long-term retention of training for the dominant and nondominant hands on the computer mouse. This allowed us to better understand whether participants truly learned the task with their nondominant hand and transferred the training or simply performed better by repeating the task for six weeks. Following approximately one year of no practice on the computer mouse by the nondominant hand, we hypothesized that, on average, the nondominant hand performance would exceed the performance from day one of the study. Additionally, we hypothesized that the one-year retention performance would not exceed the performance observed after six weeks of training, as some losses will occur if the task is not performed for a year.

## Results

### Bilateral transfer of learning effect

**Figure 1:**
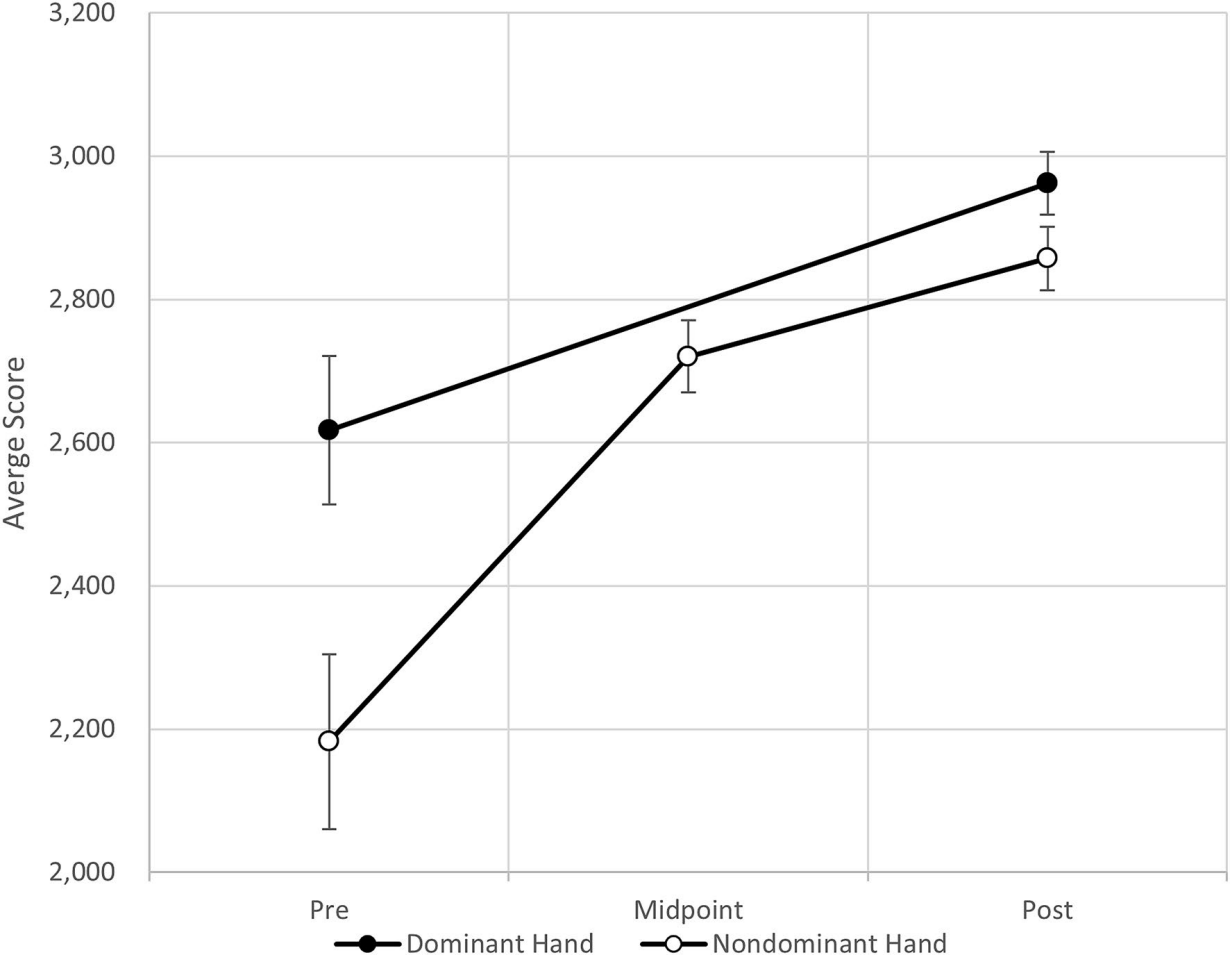
Mean values and standard errors for scores with the dominant and nondominant hands.

The first hypothesis to be tested stated that, on average, performance of the dominant hand on clicking tasks using a computer mouse would improve by training the nondominant hand for 15 min a day, five days per week, for six weeks (training period), because of bilateral transfer of learning. The average pre- and posttest scores for the dominant hand and nondominant hand are displayed in Fig. 1. Performance improved from the pre- to posttest for the dominant hand, a goal that was not emphasized during the six weeks of training, with an increase from 2617 ± 344.3 (mean score ± standard deviation) to 2962 ± 146.5, which corresponds to a 13% performance improvement on average. The nondominant hand increased from 2182 ± 405 to 2857 ± 148, which corresponds to a 31% improvement on average. When the improvements for individual participants were examined, we observed that the largest improvement to the dominant hand was 833 points (41.8%) for one participant. This participant rarely used a mouse, as they used a trackpad when working on a computer. The smallest improvement to the dominant hand was 8.2 points (0.3%) for a participant who routinely played video games with the computer mouse and had an average baseline score of 3061. The highest possible score a participant could earn in the game was 3500, so there was less opportunity to improve for this particular participant. For both the dominant and nondominant hands, a matched pairs t-test on the mean difference in participants’ scores (posttest minus pretest) was performed. For the dominant hand, the posttest mean score was significantly higher (t(df = 10) = 4.96, *p* = 0.0003) than the pretest score, with an observed increase of 345 ± 230.6 (mean score ± standard deviation). For the nondominant hand, we observed a significant improvement from the pretest to posttest (t(df = 10) = 7.07, *p* < 0.0001), with an observed mean increase of 675 ± 316.4.

The average median improvement was also analyzed to provide insight into the possible influence of extreme values, as these are known to distort the value of the mean. In addition, medians provide a better understanding of how individual participants behaved in contrast to the average of the entire group. In this analysis, the mean scores of each participant’s pretest and posttest were replaced by the median scores. These median scores were then averaged across all participants to allow for the continued use of the matched pairs t-test. The dominant hand median scores, on average, increased from 2645 ± 335.5 (mean score ± standard deviation) to 2963 ± 144.1, and the nondominant hand median scores, on average, increased from 2203 ± 403 to 2,861 ± 142.5. Median and mean analyses were performed for the bilateral transfer effect. The observed similarity in the results suggests that the data were not severely skewed and that the mean was representative of the data, as all observed median values were within 1.1% of the observed mean values.

### Nondominant hand learning curve

**Figure 2:**
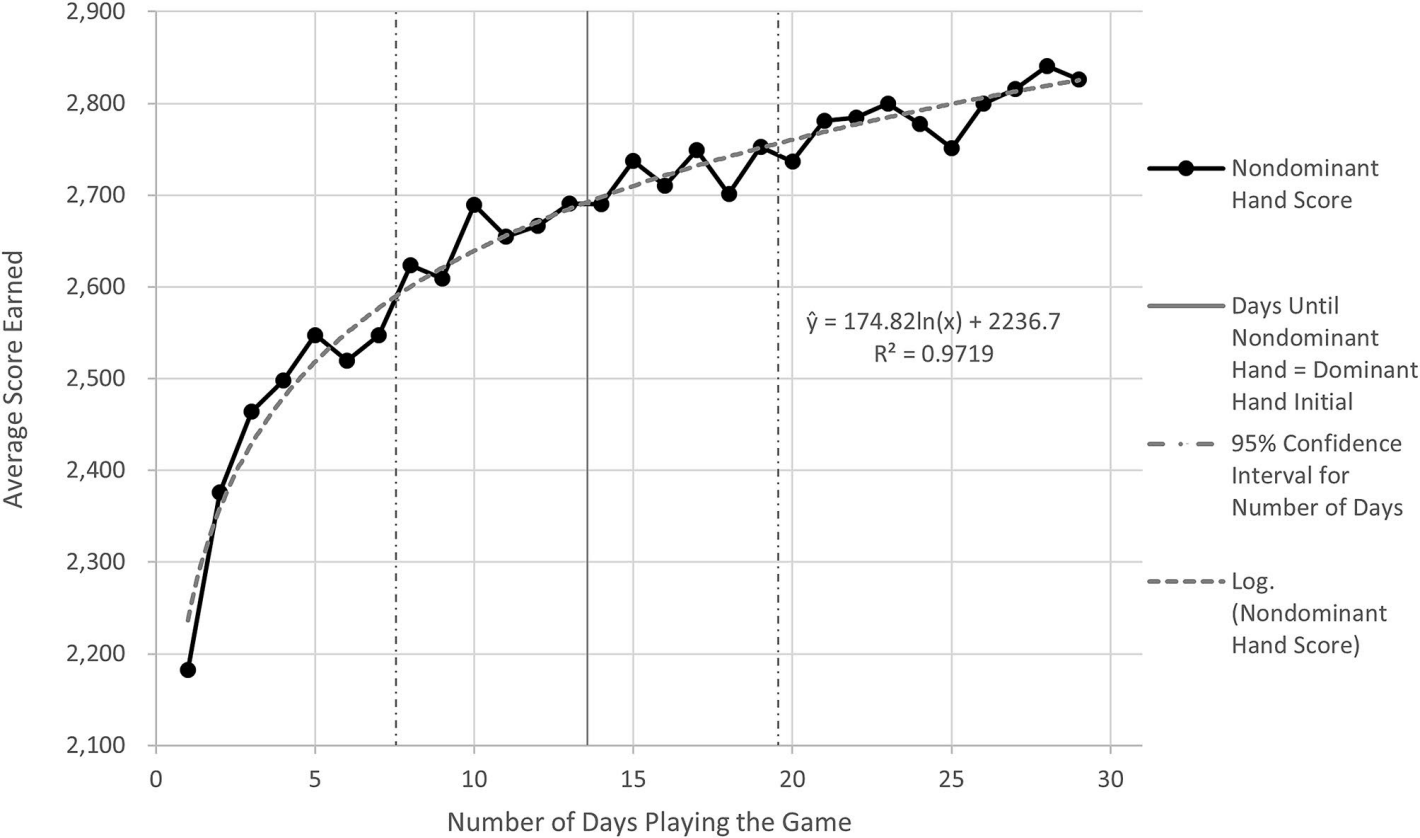
Learning curve of the nondominant hand to complete computer clicking tasks.

The second hypothesis to be tested stated that, on average, the performance of the nondominant hand would be the same as the original dominant hand performance after the training period. The resulting learning curve for the nondominant hand is illustrated in Fig. 2. The fitted curve generally follows a traditional learning curve and shows continued growth throughout before flattening at the end of the six weeks. The R^2^ value, as a goodness of fit measure of the fitted curve to the natural log curve, was 97.2%, corresponding to the amount of variability observed in the average score that can be explained by the fitted model.

All participants’ performance with their nondominant hand improved, while the scores with the nondominant hand for 91% of the participants exceeded their initial dominant hand scores. The only participant for whom this was not the case was the participant who regularly played video games with the computer mouse and started out with a high score as their baseline value. Additionally, a 95% confidence interval was constructed to estimate the average number of days it took for the nondominant hand to reach the dominant hand’s initial performance level. The confidence interval ranged from a lower bound of 6.7 days to an upper bound of 20.4 days (mean ± margin of error: 13.55 ± 6.82).

The demographic data was analyzed to determine how often the mouse was used by each participant. Six of the participants used the mouse daily, and five of the participants used the mouse weekly. Weekly users in the study tended to use laptops with trackpads rather than a mouse. Those who used the mouse daily took an average of 19.8 days for their nondominant hand to reach and remain above the performance of their dominant hand. In contrast, those who used the mouse less frequently took an average of 6.0 days for their nondominant hand to reach and remain above the performance of their dominant hand. An approximate 95% confidence interval for those who used the mouse daily ranged from a lower bound of 10.3 days to an upper bound of 29.4 days (mean ± margin of error: 19.83 ± 9.58). An approximate 95% confidence interval for those who used the mouse less frequently ranged from a lower bound of 0.05 days to an upper bound of 12.0 days (mean ± margin of error: 6.00 ± 5.95).

### Bilateral transfer of learning: evaluation of retention

**Figure 3:**
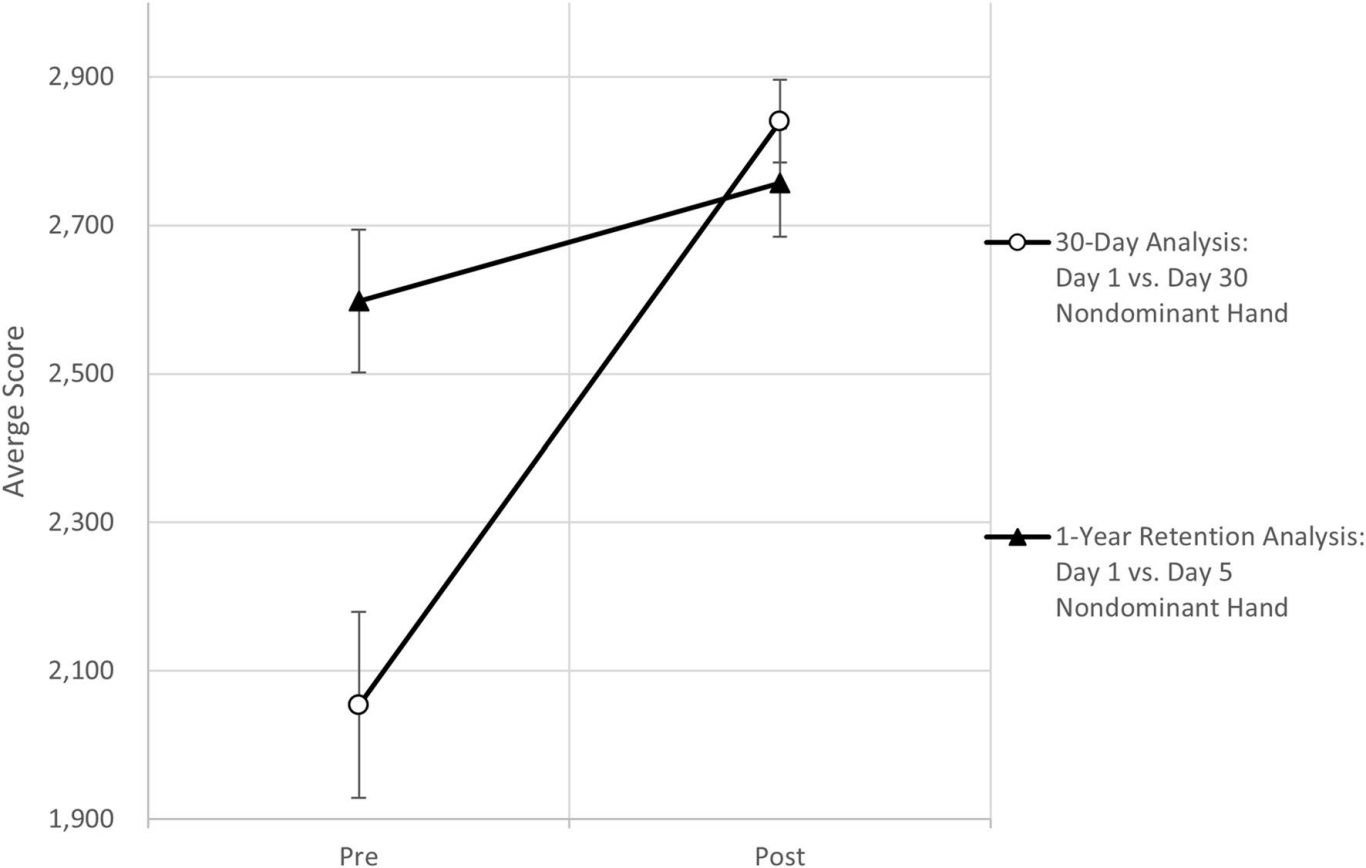
Mean values and standard errors for nondominant hand on day 1 versus day 30 and one year later on day 1 versus day 5.

The third hypothesis to be tested stated that, on average, after a year of not training the dominant or nondominant hand on the computer mouse, the participants’ nondominant hand will perform at a higher level than on the first day of the study, but not as high as after six weeks of training. The averages of the pretest and posttest scores for the nondominant hand, based on the six-week training period and the one-year retention analysis, are displayed in Fig. 3.

Eight of the original eleven study participants who completed the six-week study returned for the one-year follow-up retention study. Their nondominant hand score on day one of the six-week study was 2054 ± 354. These participants began the one-year retention study with a significantly higher (t(df = 7) = 9.51, *p* < 0.0001) average nondominant hand score of 2598 ± 273, which corresponds to a 26.5% increase compared to their original baseline score. The average nondominant hand score on day one of the retention study was significantly lower than the average score on day 30 (t(df = 7) = 3.98, *p* = 0.0026), which was consistent with our hypothesis. On average, it took the participants a mean of 12.5 days (median of 10.5 days) during the six-week training period to achieve the same level of proficiency observed on day one of the one-year retention study. After five days of playing the game approximately one year later, participants’ average nondominant hand score increased to 2757 ± 206 compared to a nondominant hand score of 2840 ± 144 on day 30 of the six-week study. This difference in scores corresponds to the participants being only 3% lower on day five of the one-year retention study than on day 30 of the six-week study. Additionally, on average, it took participants a mean of 25 days (27 median days) in the six-week training period to achieve the same level of proficiency observed on day five of the one-year retention study.

The eight returning participants began day one of the six-week study with an average dominant hand score of 2560 ± 332. Their average dominant hand score of 2888 ± 229 at the beginning of the one-year retention study was significantly higher (t(df = 7) = 7.41, *p* < 0.0001) and corresponded to a 12.8% increase over their original day-one score. The eight returning participants ended the six-week study with an average dominant hand score of 2956 ± 144 (t(df = 7) = 1.467, *p* = 0.0929), which corresponded to being only 2.4% lower approximately one year after not playing the game.

## Discussion

The results from six weeks of training with the nondominant hand suggested a significant improvement in the click speed and accuracy of both the dominant and nondominant hands, as represented by the improved scores from the pretest to the posttest. These results strongly suggested that tasks being completed in an office environment, such as clicking tasks completed with a computer mouse, can show improved performance in the dominant hand through the training of the nondominant hand. This indicates that bilateral transfer of learning exists when training from the unused hand to the traditionally used hand for computer mouse-clicking operations. Coombs and Frazer support the notion that bilateral transfer of learning shows the largest performance improvements when training the nondominant hand for the dominant hand’s benefit^27^. As discussed in the introduction, previous research has shown that bilateral transfer has been seen in a variety of applications^23–26,28^. This study differed, as it specifically addressed how bilateral transfer of nondominant hand training can be used to improve computer mouse use in the dominant hand with an application to industry. With the dominant hand showing an average 13% improvement, this equates to the participant getting the same amount of work done in 62 min less time than they had taken prior to training the nondominant hand over a typical eight-hour workday. Most people’s days are broken up by meetings, breaks, and unanticipated distractions^29^. While saving an hour per person is unlikely because of these consistent breaks in the day, the potential to meaningfully improve performance and save time each day is achievable based on the results of this study. These results suggest that training the nondominant hand may allow companies to accomplish more work via their current employees. Prepared with bilateral transfer training, employees may work more efficiently and avoid repetitive stress injuries, mitigating the need to hire additional personnel while increasing the company’s overall efficiency and output.

Using a computer mouse to click objects involves many minute movements and motor control skills of the hand and wrist to control pointer placement^30^, which are skills that have been extensively trained and refined with the dominant hand throughout people’s lifetime. Our results suggest that even a complex task that has been widely trained over many years with the dominant hand can be improved in the nondominant hand—reaching or even exceeding the initial dominant hand performance—through training. Delisle et al.’s found similar results in the nondominant hand’s performance while studying the impact of the mouse on a person’s upper extremity posture^31^. Our findings are consistent with Delisle et al.’s poststudy, which found that the performance with the nondominant hand had improved to the initial level of performance of the dominant hand for the majority of their participants^31^. Our study developed a learning curve for operating a computer mouse with the nondominant hand. The learning curve developed in our study for the nondominant hand to perform and remain above the initial performance of the dominant hand had a 95% confidence interval that ranged from 6.7 to 20.4 days for all participants. Rounding the upper end of this range to the next day, 21 days, and considering the training for 15 min a day, the total training time equated to 5.25 h. Considering the grand scheme of an average of 2000 h of work in a year, 5.25 h is a short period of training time to increase the performance of the traditionally used hand.

By examining how the participants’ mouse experience affected the learning of the nondominant hand, we found that less experienced users (minimal use in a week) were able to learn, on average, fourteen days faster with their nondominant hand than the experienced users (daily use). There was a substantial difference, as the experienced users took, on average, 19.8 days for their nondominant hand to reach and remain above the performance of their dominant hand. In contrast, the less experienced users took, on average, only 6.0 days. However, when looking at the participants’ initial scores with their dominant hand, this difference is no longer surprising. The participants who used the mouse less frequently did not score as well initially with their dominant hand. This represented slower and less accurate clicking performance for less experienced participants. Based on the learning curve from this study, that is, Fig. 2 of the results, the scores rapidly increased in the first ten days before beginning to gradually level off. For the less experienced users, the nondominant hand quickly adapted and performed better than the dominant hand. The participants who used the mouse daily had originally scored, on average, 16% higher with their dominant hand than the weekly mouse users. Therefore, for the daily mouse users, it took longer for the performance of the nondominant hand to achieve those same scores. Baher and Westerman found that in specific Photoshop tasks, expert users took longer to complete tasks than novice users because rather than using the parts built into the interface to make their work faster, they used more familiar approaches that took longer^32^. The experienced users of the mouse may have more familiar mouse patterns that involve more refined, minute movements that their nondominant hand takes additional time to learn.

The use of a computer mouse by one hand for extended periods of time can put people at risk of cumulative trauma disorders such as carpal tunnel syndrome. Since the use of a mouse requires the wrist to be in extension for long periods of time performing multidimensional movements, this causes carpal tunnel pressure to increase^33^. However, research has shown that when the mouse was used by the nondominant hand, wrist extension was reduced in the nondominant hand^31^. Successful training of the nondominant hand could lead to a reduction in carpal tunnel syndrome and other repetitive force injuries that are currently seen in the dominant hand from overuse. Another way carpal tunnel syndrome and other repetitive force injuries could be reduced is by switching which hand uses the mouse with throughout the day. Research has shown that rest is an easy way to prevent fatigue^34,35^. Using the nondominant hand to complete computer mouse operations will allow workers to switch hands during the day to reduce load and give the hands and wrists time for rest and repair. Rather than frequent breaks to prevent fatigue^36^, in which there is no production from the employee, the employees within companies can alternatively rest the dominant hand while continuing to work with the nondominant hand. Rotating hand use is also similar to a job rotation program in the sense that it changes the posture the workers are in for long periods of time. Worker rotation programs have been an effective industry practice for many years and have been shown to prevent ergonomic injuries, as they reduce static postures that are being assumed by employees for long periods of time^37^. Implementing hand rotation based on this successful practice, which is often used in manufacturing or service settings, may reduce the risk of injury in office environments.

Some may argue that the clicking game is not representative of work and cannot represent an industry task. However, many tasks in the work environment involve clicking with a computer mouse, as a study found that the average computer user clicks 7400 times each week while working on the computer for an average of 12.4 h a week^38^. This is an average of 9.95 clicks/min, which equates to just over a click every six seconds, while working on the computer in the workplace^38^. Whether it is opening software, changing cells in Excel, switching tabs, or closing browsers, clicking is one of the most common computer tasks. The game aspect was used to foster the participants' natural motivation to perform better and limit variability in the participants' motivation to improve^39^. The game is representative of the same sensorimotor applications people use in their job, making it an appropriate tool to judge the performance of industry tasks.

The results from the one-year retention study suggested that the dominant and nondominant hands retained a significant amount of click speed and accuracy after not being used for approximately a year, as represented by the improved scores from day one of the six-week study to day one of the retention study. These results strongly suggested that the nondominant hand learned the skills to control the speed and accuracy of the mouse movements and did not simply improve because of repetition. The results by Fleishman et al. supported the notion that after two years of not practicing complex, continuous control, perceptual-motor skills, the performance initially decreased, as participants refamiliarized themselves with the task, yet participants recovered learning when they had gained proficiency in the initial training^40^. The results of the one-year retention study also supported the notion that the dominant hand experienced bilateral transfer of training as the dominant hand performance after not training for a year was significantly higher than day one of the original study, and only 2.4% lower, on average, then at the end of the six-week training. Purdy el al. found that the level of proficiency in a task that took ten days in the original training to achieve was reached within three days after not practicing for one year^41^. This coincided with our one-year retention study results, which found that after not completing the task for approximately a year, the participants were able to reach the level of proficiency they had on day 25 of the original study after five days of practicing.

## Conclusion and future directions

Operating a computer mouse is a daily task in the workplace and is primarily completed with the right hand due to workstation setup, which results in the formation of a hand dominance. By consistently using the mouse with the same hand over a prolonged period of time, people can develop repetitive use injuries, such as carpal tunnel syndrome, and performance on the computer can come to a natural stagnation. This study examined training the nondominant hand on a computer mouse during a typical work week and observed improvements in the dominant hand performance due to bilateral transfer of learning. Additionally, the performance and learning curve of the nondominant hand was also analyzed, and the nondominant hand could be trained to the initial performance of the dominant hand. Last, when the nondominant hand was not trained for a prolonged period, the performance decreased but remained significantly higher than prior to the original training; that is, the nondominant hand can be rapidly retrained to the previous final performance in a few days, showing that the skill was truly trained and obtained. Thus, our results show that training the nondominant hand on the computer mouse allowed for improved performance with both the dominant and nondominant hands while also providing safer, more sustainable, and more achievable work in a multitude of economies.

Due to the length of the study, finding volunteers to participate was difficult, leaving our study with a smaller sample size than originally planned. However, because the results from the nonparametric statistical tests were of the same magnitude as the parametric tests, we were confident in using all the parametric analysis techniques. Additionally, because of participants’ changing schedules, a few days were missed by the participants. However, these few missed days were deemed not statistically deteriorating, as every participant played at least three days in each week, and the analysis was performed based on the number of days of training, not on specific days of training. Third, we evaluated the scores collected, which were based on clicking accuracy and speed of the user completing the task. However, we could not evaluate whether there were more significant improvements in accuracy, speed, or both. Some may argue that the increased scores by the dominant hand could have resulted from the participants developing better strategies for performing the task. However, the game had very little strategy that could be employed because to obtain a high score, the participant must go as fast as they can while clicking the centers. If they follow a strategy of clicking the centers, they do not obtain very good scores, and the same is true if they go only for speed. The participants needed to improve both accuracy and speed in order to obtain higher scores. Additionally, in four of the levels, the targets showed up in random spots on the screen; for those levels, a strategy cannot be formed. On the levels where the targets were worth the most points, those targets must have been clicked before they left the screen, so those levels were also purely performance based. The only level where a strategy could have been used in the game was on the last level where there was a 6 × 8 grid of targets. How a participant goes about clicking the targets in that grid affects the speed, which affects the points. If the participants did not originally go from top to bottom, left to right, or vice versa, then this would be a strategy that could have developed. However, within the first five attempts on the first day, all participants were clicking using one of those patterns, which indicated that they had already formed their strategy within this first day. Due to the game having no strategy, we feel our results prove bilateral transfer; however, in future studies on bilateral transfer, the game should be played with the dominant hand until the scores level off on the first day to affirm the results. This would prove that the dominant hand had a strategy and that the improvements were solely from bilateral transfer. Last, all tasks performed in the game involved clicking, but there are other types of computer tasks, such as dragging and placing, that add more complexity and minute movements with the hand, which could take longer to train. Therefore, we can comment only on applications involving clicking tasks with the computer mouse. In future studies, these limitations should be considered.

Existing research has shown that handedness is easier to control when training people when they are young^1^. Future research is needed to examine whether the learning curves change when training to use the mouse with both hands happens at a young age. More research is also needed to examine whether training both hands from a young age helps prevent injury while improving human performance. Research is needed to identify whether dragging, placing, and other mouse movements with the nondominant hand influence the learning curve. A nondominant hand training program should be implemented across a variety of industries to understand if the impacts on performance and injury reduction are stronger in some industries than in others.

Researchers have measured and evaluated bilateral transfer following several different approaches, such as electromyogram signal recordings^42^, mirror visual feedback^43^, electronic mirror-drawing apparatuses^44^, perceptual motor tasks involving mirrors^45^, perceptual tasks involving different sensory modalities^46^, electrophysiological indices^47^, or scaled questionnaires and video tapes^48^. Other researchers have demonstrated bilateral transfer using functional magnetic resonance imaging (fMRI). In 2016, Uggetti et al^49^ investigated the association of specific brain activation patterns using fMRI with bilateral transfer while completing a nine hole peg test with nondominant hand and dominant hand training and found significant fMRI activation before and after training, particularly in movement control, coordination and working memory areas. In 2007, twenty-five participants were analyzed using fMRI while they performed a visuomotor adaption joystick task to position a cursor in a target circle, which appeared on a screen in one of four locations^50^. In terms of endpoint accuracy, they found a transfer of sensorimotor function from the dominant arm to the nondominant arm^50^. In future work, the use of fMRI will assist in widening the investigation area in terms of the brain activation areas associated with bilateral transfer while performing this kind of task.

## Methodology

### Participants

Eleven healthy right-handed computer mouse users (four males and seven females) completed the study. The age range was 20–58 years, with an average age of 36.7 years. In addition, the participants were widely diverse in occupation (one business owner, three engineers, one grocery store clerk, two students, one elementary school teacher, one high school teacher, one manager, and one supervisor). Although three participants ate, wrote, and threw with their left hand, all used the computer mouse with their right hand. To minimize sampling bias, a variety of recruitment strategies were used to reach a larger participant pool that accounted for diverse backgrounds. The study was considered exempt by Iowa State University’s Institutional Review Board (IRB). Formal consent was also deemed exempt by IRB, but all participants were read a description of the study and informed that their participation was voluntary. All methods were performed in accordance with relevant guidelines and regulations.

### Experimental procedure: six-week training period

Demographic characteristics were collected to gain information about the participants, including which hand they regularly used to complete tasks and how often. Next, participants completed the Minnesota Dexterity Placing and Turning test three times with their dominant hand and three times with their nondominant hand to record baseline dexterity prior to completing tasks for the study. Following the dexterity tests, the participants played a mouse-clicking game that can be found at http://www.roomrecess.com/mobile/ClickSpeed/play.html. Playing the game five times equates to approximately 15 min, which coincided with the training time included in our hypotheses. The game was suitable for the purpose of our study because it requires a variety of clicking motions that people often employ when completing tasks on the computer in industry settings. Additionally, this game was selected because of its competitive scoring, which provided a more natural incentive for the participants to consistently do well; the higher the score was, the better the player performed. The scoring in the game is based on the accuracy of clicking the center of the target and the speed at which the participants clicked. There were a total of 119 targets in the game. The point value for clicking the center of the targets varied between 10 and 30 points. If the centers were not clicked, the participants still received one, three, or five points depending on how close to the center they were. Additionally, half the levels in the game have bonus points that began at 100 points and dropped by approximately nine points per second. The most points were available on the last level with 48 targets. The bonus points began at 1000 points and dropped by approximately nine points per second. The bonus points encouraged participants to click fast, and the added points for clicking the center of the target encouraged accuracy.

The participants played the game five times with their dominant hand, and the score they received was recorded after each game. The average of these five scores served as a baseline performance measure for each participant’s dominant hand. The primary mouse key was then changed from left to right. Changing the primary key adjusted the mouse to mirror how the dominant hand uses the mouse with the index finger clicking and the middle finger resting on the secondary key. This allowed for the bilateral transfer effect to be analyzed as the hands performed the same task with the same motions. The game was played five times with the nondominant hand, and the score they received was recorded after each game to identify a baseline performance for each participant’s nondominant hand.

The participants played the game five times each day for the next four days (for a total of five days in week 1) with their nondominant hand. Participants then took two days off to simulate a weekend, as seen in most working environments. For the next five weeks (a total of six weeks), the participants played five times for five straight days and took two days off. Due to participants’ changing schedules, a few days were missed by all participants. However, this did not negatively affect the statistical analysis and reliability of the results, as every participant played at least three days in each week, and the analysis was performed based on the number of days of training, not on specific days of training. All participant training lasted six weeks, but the number of days each participant played the game five times varied. Six participants completed 29 out of 30 days, two completed 28 out of 30, and one completed 26, 25, and 23 out of 30 days. On the final day, the participants played five times with their dominant hand to determine if their performance had improved, despite not playing the game with that hand since the first day.

### Experimental procedure: one-year retention study

Participants were contacted approximately one year after completing the original six-week study and asked to participate in playing the game with their nondominant hand five times per day for five consecutive days to evaluate the retention of their nondominant hand skills. Eight of the eleven participants played the game with their nondominant hand five times for five consecutive days. The other three participants were unavailable for the follow-up experiment. Additionally, the participants played the game with their dominant hand five times on the first day to evaluate their baseline performance after not playing the game for approximately a year.

### Statistical analysis

**Figure 4:**
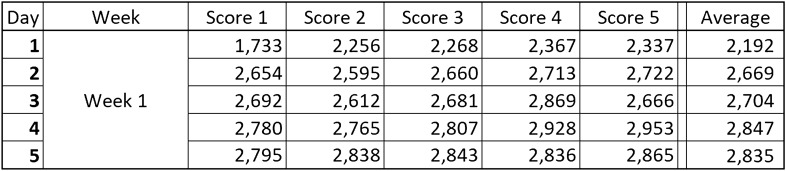
Typical week for each participant. Five days, with five scores each day, which were then averaged for analysis.

Statistical analyses were computed using R Version 3.6.1^51^. Over six weeks, each participant performed the experiment five times a day for five consecutive days before taking a two-day break. This resulted in six blocks of five measurements each for the ideal participant. The five obtained scores within each day were averaged for each participant, yielding five means collected for each week for each participant. An example of how a participant’s data for one week might look is shown in Fig. 4.

To answer the hypotheses of interest in this study, we used the data in three distinct ways. For hypotheses one and two, we analyzed the difference in the average scores achieved from the beginning to the end of the study by calculating the difference between the pretest and posttest average scores for each of the eleven participants. These differences were then averaged across all participants to test if the mean difference was greater than zero, implying that all participants improved, on average, over the six-week period. A matched pairs t-test was used to analyze the mean difference, but because of the small sample size (n = 11) and potential lack of normality of the data, a Wilcoxon signed rank test was also performed as a follow-up. The Wilcoxon signed rank test is a nonparametric test that does not rely on the sample means following, at least approximately, a normal distribution. In all cases, the conclusions from the Wilcoxon signed rank test was the same as that from the matched pairs t-test, namely, to reject the null hypothesis. Although not identical, p-values from each test were of similar magnitude. To calculate the number of days until the nondominant hand achieved performance levels similar to the initial level of the dominant hand (hypothesis two), the number of days for all participants was averaged, and a 95% confidence interval was constructed. For hypothesis three, we analyzed the difference in the average scores achieved between the timepoints of interest for the eight participants who returned for the one-year retention study. Despite the further decrease in sample size, the results from a matched pairs t-test and the Wilcoxon signed rank test showed only small differences with respect to the strength of evidence found in the data.
